# Supplementary material for: Genetic Susceptibility to Incisional Hernia Evaluation of Hernia Polygenic Risk Scores
Source: medRxiv. 2026 Jun 11:2026.06.10.26355374. Preprint. [Version 1] doi: 10.64898/2026.06.10.26355374 (PMC13278284; doi:10.64898/2026.06.10.26355374)
Supplement: Supplementary file 1 [file NIHPP2026.06.10.26355374V1-supplement-1.pdf]

medRxiv preprint doi: https://doi.org/10.64898/2026.06.10.26355374; this version posted June 11, 2026. The copyright holder for this preprint (which was not certified by peer review) is the author/funder, who has granted medRxiv a license to display the preprint in perpetuity.
It is made available under a CC-BY 4.0 International license .

Supplementary Material

Incisional Hernia Phenotyping

Incisional hernia was ascertained using administrative and billing diagnosis and procedure codes from the electronic health record, including ICD-9-CM, ICD-10-CM, ICD-9-PCS, ICD-10-PCS, and Current Procedural Terminology (CPT) codes. Code lists used to define ventral/incisional hernia diagnosis and repair are provided in the Supplementary Data.

Patients were excluded from the at-risk cohort if they had evidence of a preexisting ventral or incisional hernia, defined as a ventral/incisional hernia diagnosis code occurring on or before their index abdominal operation. Patients were also excluded if the index operation was a ventral or incisional hernia repair, defined by the presence of any ventral/incisional hernia repair code on the date of their index operation. Primary ventral hernia codes could not be reliably distinguished from incisional hernia codes due to overlap in coding practices, subsequently, ventral and incisional hernia codes were analyzed together. Patients with umbilical hernia diagnosis or repair alone were not excluded.

The primary outcome was postoperative ventral/incisional hernia, defined as the first occurrence after the index operation of either a ventral/incisional hernia diagnosis code or a ventral/incisional hernia repair code. Because ICD-10-PCS and newer CPT repair codes classify hernia repair by size rather than type, repair codes that could represent ventral/incisional or umbilical hernia repair were required to have a concurrent ventral/incisional hernia diagnosis code to be classified as the primary outcome.

Postoperative ventral/incisional hernia was defined as the first occurrence of the following:

- An ICD-9-CM diagnostic code for ventral/incisional hernia
- An ICD-10-CM diagnostic code ventral/incisional hernia
- An ICD-9-PCS repair code for ventral/incisional hernia
- A CPT repair code specific for ventral/incisional hernia
- An ICD-10-PCS repair code for ventral/incisional/umbilical hernia repair with an ICD-10-CM diagnostic code for ventral/incisional hernia
- A CPT repair code for ventral/incisional/umbilical hernia repair with either an ICD-9-CM or ICD-10-CM diagnostic code for ventral/incisional hernia

Outcome status was assessed within a five-year follow-up window from the index operation date. Patients were censored at the earliest of death, a subsequent non-hernia abdominal operation, date of last follow-up if less than five years after the index operation, or five years after the index operation.

medRxiv preprint doi: https://doi.org/10.64898/2026.06.10.26355374; this version posted June 11, 2026. The copyright holder for this preprint (which was not certified by peer review) is the author/funder, who has granted medRxiv a license to display the preprint in perpetuity.
It is made available under a CC-BY 4.0 International license .

Table S1: Summary of Number of Cases, Controls, and Effective Sample Size for Input Summary Statistics, Stratified by Phenotype and Population

Phenotype	Population	Cases	Controls	Effective Sample Size
Abdominal	AFR	16168	143674	56991
	AMR	8344	80501	29338
	EAS	128	2581	488
	EUR	152926	1281340	528423
	MID	224	1375	770
Diaphragmatic	AFR	4635	157207	17991
	AMR	2556	87512	9924
	CSA	660	7837	2435
	EAS	88	2581	340
	EUR	73070	1288859	274401
	MID	139	1375	505
Femoral	EUR	2446	790529	9751
Inguinal	AFR	7284	158627	27556
	AMR	3387	87335	12860
	CSA	297	7837	1145
	EAS	1664	177062	6594
	EUR	93719	1297379	347012
	MID	70	1375	266
Umbilical	AFR	2537	163798	9992
	AMR	1422	89487	5595
	CSA	90	7837	356
	EUR	27318	1320619	106859
Ventral	AFR	1258	166521	4994
	AMR	793	90972	3144
	CSA	58	7837	230
	EUR	21245	1331234	83473

medRxiv preprint doi: https://doi.org/10.64898/2026.06.10.26355374; this version posted June 11, 2026. The copyright holder for this preprint (which was not certified by peer review) is the author/funder, who has granted medRxiv a license to display the preprint in perpetuity.
It is made available under a CC-BY 4.0 International license .

Table S2: Distribution of hernia polygenic risk scores, stratified by hernia subtype

Phenotype	Polygenic Risk Score	Mean PRS Cases	Mean PRS Controls	P-Value
Any Hernia	Abdominal PRS	0.13	0.02	< 0.001
	Ventral PRS	0.06	0.02	< 0.001
Ventral Hernia	Abdominal PRS	0.18	0.04	< 0.001
	Ventral PRS	0.11	0.03	< 0.001

medRxiv preprint doi: https://doi.org/10.64898/2026.06.10.26355374; this version posted June 11, 2026. The copyright holder for this preprint (which was not certified by peer review) is the author/funder, who has granted medRxiv a license to display the preprint in perpetuity.
It is made available under a CC-BY 4.0 International license .

Table S3: Clinical and Demographic Characteristics of Patients Undergoing Abdominal Surgery, Stratified by Incisional Hernia Status

Polygenic Risk Score	Ancestry	Odds Ratio (95% CI)	P-Value
PRSCSx_CommonFacto	EUR	1.18 (1.11 to 1.25)	< 0.001
	AFR	1.07 (0.99 to 1.17)	0.087
	SAS	1.34 (0.84 to 2.15)	0.219
	EAS	1.07 (0.36 to 3.35)	0.91
	AMR	1.45 (1.03 to 2.06)	0.035
	Other	1.07 (0.59 to 1.93)	0.823
PRSCSx_Abdominal	EUR	1.17 (1.11 to 1.23)	< 0.001
	AFR	1.09 (1.01 to 1.18)	0.029
	SAS	1.79 (1.09 to 2.97)	0.023
	EAS	0.41 (0.13 to 1.23)	0.116
	AMR	1.36 (0.96 to 1.92)	0.081
	Other	0.93 (0.51 to 1.7)	0.82
PRSCSx_Ventral	EUR	1.1 (1.04 to 1.16)	< 0.001
	AFR	1.02 (0.94 to 1.1)	0.707
	SAS	1.66 (1.00 to 2.77)	0.051
	EAS	0.83 (0.29 to 2.36)	0.731
	AMR	1.26 (0.90 to 1.77)	0.181
	Other	1.49 (0.83 to 2.73)	0.188

medRxiv preprint doi: https://doi.org/10.64898/2026.06.10.26355374; this version posted June 11, 2026. The copyright holder for this preprint (which was not certified by peer review) is the author/funder, who has granted medRxiv a license to display the preprint in perpetuity.
It is made available under a CC-BY 4.0 International license .

Table S4: Association of PRS-Ventral with Incisional Hernia When Adjusting for Clinical Risk Factors

		Hazard Ratio (95% CI)	P-Value
Age (Per Year Increase)		1.02 (95% CI: 1.01 - 1.02)	< 0.001
Sex	Female	Ref	
	Male	0.99 (95% CI: 0.84 - 1.16)	0.897
BMI (kg/m^2, Per Unit Increase)		1.03 (95% CI: 1.02 - 1.04)	< 0.001
Smoking	No	Ref	
	Yes	1.38 (95% CI: 0.78 - 2.45)	0.266
Perioperative SSI	No	Ref	
	Yes	2.42 (95% CI: 1.84 - 3.17)	< 0.001
Index Surgery Type	Bariatric	Ref	
	Colorectal	4.93 (95% CI: 3.33 - 7.28)	< 0.001
	Gynecologic	1.63 (95% CI: 1.05 - 2.54)	0.029
	Hepatobiliary	2.57 (95% CI: 1.72 - 3.83)	< 0.001
	Non-Incisional Hernia Repair	1.64 (95% CI: 1.03 - 2.61)	0.037
	Transplant	2.46 (95% CI: 1.62 - 3.74)	< 0.001
	Upper Gastrointestinal	1.30 (95% CI: 0.77 - 2.19)	0.328
	Urologic	2.13 (95% CI: 1.40 - 3.24)	< 0.001
	Vascular	2.04 (95% CI: 1.08 - 3.83)	0.028
PRS-Ventral		1.10 (95% CI: 1.01 - 1.18)	0.019

medRxiv preprint doi: https://doi.org/10.64898/2026.06.10.26355374; this version posted June 11, 2026. The copyright holder for this preprint (which was not certified by peer review) is the author/funder, who has granted medRxiv a license to display the preprint in perpetuity.
It is made available under a CC-BY 4.0 International license .

Table S5: Association of PRS-Abdominal with Incisional Hernia When Adjusting for Clinical Risk Factors

		Hazard Ratio (95% CI)	P-Value
Age (Per Year Increase)		1.02 (95% CI: 1.01 - 1.02)	< 0.001
Sex	Female	Ref	
	Male	1.00 (95% CI: 0.85 - 1.18)	0.995
BMI (kg/m^2, Per Unit Increase)		1.03 (95% CI: 1.02 - 1.04)	< 0.001
Smoking	No	Ref	
	Yes	1.37 (95% CI: 0.78 - 2.43)	0.276
Perioperative SSI	No		
	Yes	2.42 (95% CI: 1.85 - 3.18)	< 0.001
Index Surgery Type	Bariatric		
	Colorectal	4.94 (95% CI: 3.34 - 7.29)	< 0.001
	Gynecologic	1.65 (95% CI: 1.06 - 2.57)	0.026
	Hepatobiliary	2.58 (95% CI: 1.73 - 3.85)	< 0.001
	Non-Incisional Hernia Repair	1.62 (95% CI: 1.01 - 2.57)	0.043
	Transplant	2.46 (95% CI: 1.62 - 3.74)	< 0.001
	Upper Gastrointestinal	1.29 (95% CI: 0.77 - 2.19)	0.334
	Urologic	2.13 (95% CI: 1.41 - 3.24)	< 0.001
	Vascular	2.03 (95% CI: 1.08 - 3.83)	0.028
PRS-Abdominal		1.12 (95% CI: 1.04 - 1.20)	0.003

Table S6: Brier score estimates and probability of equivalence

Model	Brier Score	Brier Score 95% CI	Model Difference	Model Difference 95% CI	Probability of Equivalence
Clinical	0.047	0.046-0.048	< 0.001	<0.001 - < 0.001	100%
Clinical + PRS-Hernia	0.047	0.046-0.048			
Clinical	0.047	0.046-0.048	< 0.001	<0.001 - < 0.001	100%
Clinical + PRS-Ventral	0.047	0.046-0.048			
Clinical	0.047	0.046-0.048	< 0.001	<0.001 - < 0.001	100%
Clinical + PRS-Abdominal	0.047	0.046-0.048			

medRxiv preprint doi: https://doi.org/10.64898/2026.06.10.26355374; this version posted June 11, 2026. The copyright holder for this preprint (which was not certified by peer review) is the author/funder, who has granted medRxiv a license to display the preprint in perpetuity.
It is made available under a CC-BY 4.0 International license .

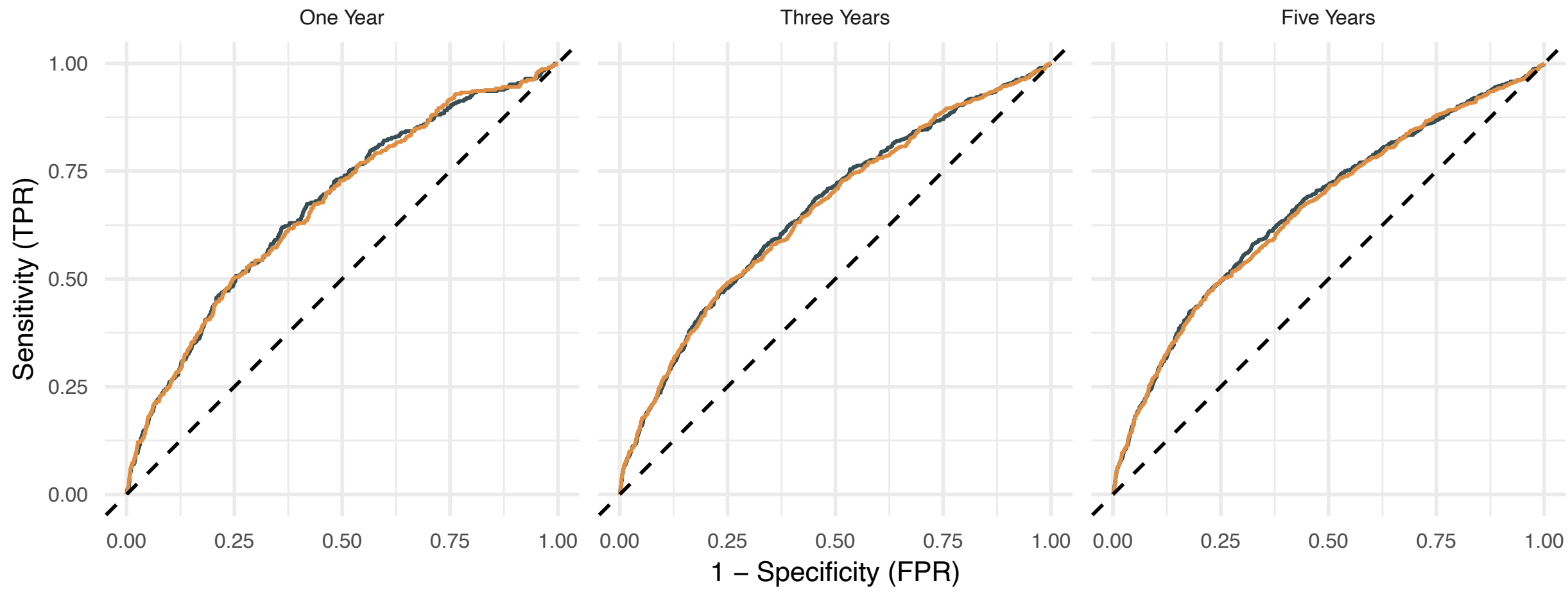

Model	AUROC (95% CI)	$P_{Equivalent}$
Clinical	0.670 (0.661-0.678)	0.999
Clinical PRS-Ventral	0.672 (0.664-0.680)	

Model	AUROC (95% CI)	$P_{Equivalent}$
Clinical	0.657 (0.650-0.664)	1.0
Clinical PRS-Ventral	0.660 (0.653-0.667)	

Model	AUROC (95% CI)	$P_{Equivalent}$
Clinical	0.660 (0.653-0.666)	1.0
Clinical PRS-Ventral	0.664 (0.658-0.670)	

Model — Clinical + PRS-Ventral — Clinical

Figure S1: Time-dependent receiver operating characteristic curves for incisional hernia prediction at one, three, and five years. ROC curves compare discriminative performance of a clinical-only model versus a model augmented with PRS-Ventral (Clinical + PRS-Ventral) at each time horizon. Tables below each panel report the Area Under the ROC Curve (AUROC) with 95% confidence intervals and the probability of equivalence (P-Equivalent) between models. The two models demonstrated near-identical discrimination across all time points (AUROC ~0.67–0.68), with no meaningful improvement conferred by the addition of PRS-Ventral.

medRxiv preprint doi: https://doi.org/10.64898/2026.06.10.26355374; this version posted June 11, 2026. The copyright holder for this preprint (which was not certified by peer review) is the author/funder, who has granted medRxiv a license to display the preprint in perpetuity. It is made available under a CC-BY 4.0 International license .

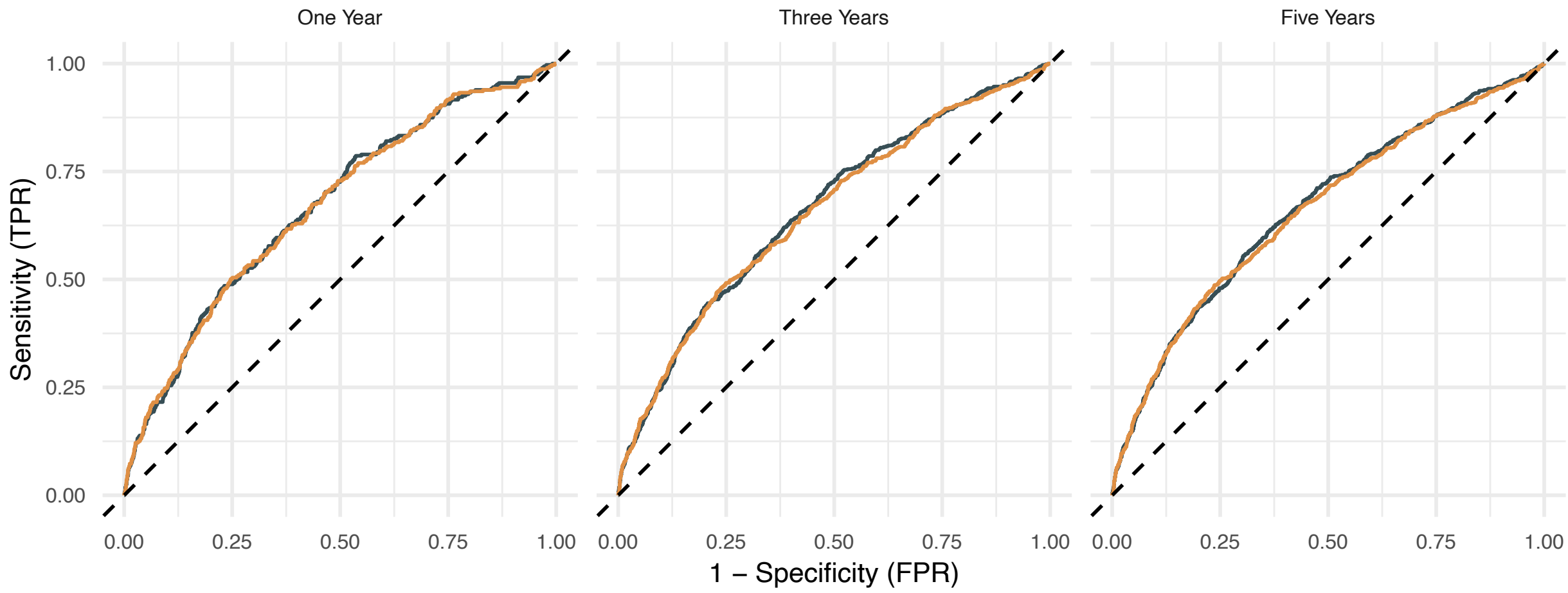

Model	AUROC (95% CI)	$P_{Equivalent}$
Clinical	0.670 (0.661-0.678)	0.999
Clinical PRS-Abdominal	0.672 (0.664-0.680)	

Model	AUROC (95% CI)	$P_{Equivalent}$
Clinical	0.657 (0.650-0.664)	0.999
Clinical PRS-Abdominal	0.662 (0.656-0.669)	

Model	AUROC (95% CI)	$P_{Equivalent}$
Clinical	0.660 (0.653-0.666)	1.0
Clinical PRS-Abdominal	0.664 (0.658-0.670)	

Model — Clinical + PRS-Hernia — Clinical

Figure S2: Time-dependent receiver operating characteristic curves for incisional hernia prediction at one, three, and five years. ROC curves compare discriminative performance of a clinical-only model versus a model augmented with PRS-Abdominal (Clinical + PRS-Abdominal) at each time horizon. Tables below each panel report the Area Under the ROC Curve (AUROC) with 95% confidence intervals and the probability of equivalence (P-Equivalent) between models. The two models demonstrated near-identical discrimination across all time points (AUROC ~0.67–0.68), with no meaningful improvement conferred by the addition of PRS-Abdominal.
